# Use of imiquimod in 21 patients with molluscum contagiosum: better results in specific areas^؆^

**DOI:** 10.1016/j.abd.2025.501142

**Published:** 2025-07-07

**Authors:** John Verrinder Veasey, Bruna Cavaleiro de Macedo Souza, Rebeca Naomi Shida, Guilherme Camargo Julio Valinoto

**Affiliations:** a Dermatology Clinic, Hospital da Santa Casa de São Paulo, São Paulo, SP, Brazil; b Discipline of Dermatology, Faculty of Medical Sciences, Santa Casa de São Paulo, São Paulo, SP, Brazil

*Dear Editor,*

There are several therapeutic options for Molluscum contagiosum (MC), including physical treatments (electrocautery, curettage, cryotherapy), chemical treatments (potassium hydroxide, imiquimod) and even waiting for spontaneous resolution.^1^, ^2^, ^3^, ^4^ Imiquimod (IMQ) is a topical immunomodulator that acts by stimulating the innate and adaptive immune pathways. Initially approved for the treatment of anogenital warts, actinic keratosis, and superficial basal cell carcinoma, there are other off-label indications in both benign and malignant dermatological diseases due to its potential antiviral, antitumor, and immunoregulatory effects.^5^, ^6^

A retrospective study was conducted analyzing medical records of patients with MC treated at a dermatology clinic in a public tertiary hospital in São Paulo, Brazil, from March 2016 to March 2024, evaluating: (i) The number of patients treated with IMQ, (ii) Therapeutic response, and (iii) Side effects presented by patients. Of the 1,256 patients treated in eight years, IMQ was indicated in only 21 (Table 1); the gender ratio was similar (11 women, 10 men), and only three (14.28%) were over 18 years old. All treated lesions were located on the face (8, 38.09%) or in the anogenital region (13, 61.91%). All patients used a similar dose to that used for the treatment of anogenital warts (three times a week) for a period of four to 33 weeks. Regarding the therapeutic response, 15 patients showed complete resolution (Figure 1, Figure 2, Figure 3), three showed partial response and three others showed no response. Patients without response used the product for four weeks, whereas those with partial response used it for four to eight weeks and all patients with complete response used the product for eight weeks or more.

**Table 1:** Characteristics of 21 patients with molluscum contagiosum treated with imiquimod.

Patient	Sex	Age	Location	Treatment duration (weeks)^a^	Response to treatment	Adverse effects
1	Female	4	Genital	8	Partial	-
2	Female	11	Genital	4	Total	-
3	Male	5	Face	4	Total	-
4	Male	5	Face	28	Total	-
5	Female	5	Face	20	Total	-
6	Male	8	Face	8	Total	Erythema
7	Female	12	Face	4	Absent	-
8	Male	55	Genital	8	Total	-
9	Female	21	Anal	4	Absent	Bacterial infection
10	Female	48	Genital	4	Partial	-
11	Male	8	Face	8	Total	-
12	Male	5	Face	4	Total	-
13	Male	6	Face	4	Partial	-
14	Male	8	Genital	4	Total	Erythema
15	Male	6	Genital	4	Absent	-
16	Female	3	Anal	16	Total	-
17	Male	4	Genital	12	Total	-
18	Female	7	Anal	33	Total	-
19	Female	5	Anal	8	Total	Bacterial infection
20	Female	3	Anal	28	Total	-
21	Female	7	Anal	4	Total	Erythema

a All patients used imiquimod three times a week; (-) Absent.

**Figure 1:**
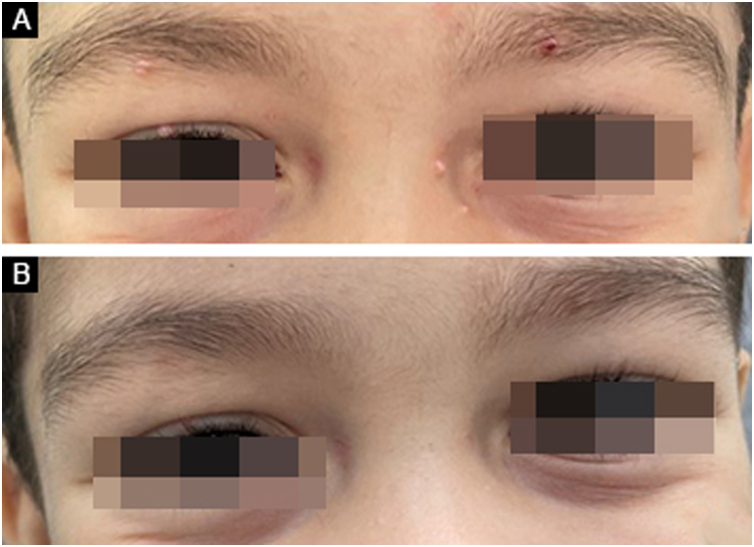
Patient with molluscum contagiosum around the eyes treated with imiquimod. (A) Before treatment; (B) Clinical outcome after four weeks.

**Figure 2:**
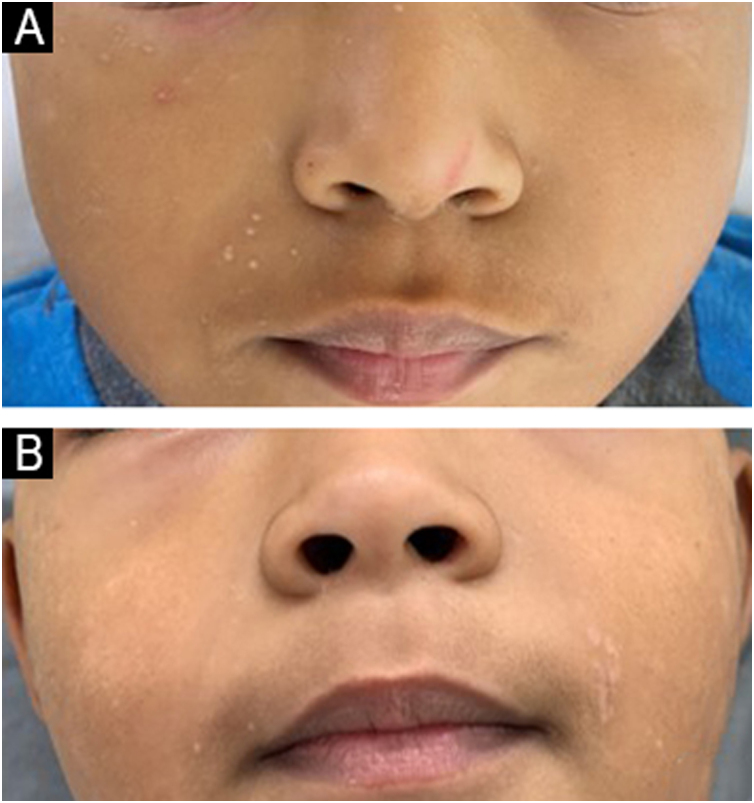
Patient with molluscum contagiosum on the right side of the face treated with imiquimod. (A) Before treatment; (B) Clinical outcome after 28 weeks.

**Figure 3:**
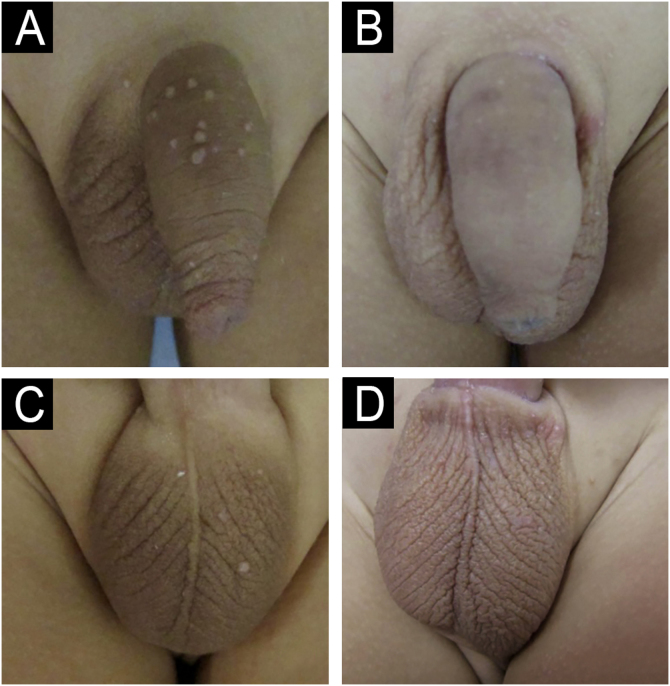
Patient with molluscum contagiosum in the genital region treated with imiquimod. (A‒B) Before treatment; (C‒D) Clinical outcome after 12 weeks.

Given the number of patients treated during the analyzed period, it is clear that IMQ is not the first-choice treatment at the institution where the study was conducted. One of the reasons is that the study was conducted in a public hospital that does not provide IMQ free of charge to patients, and few of them can afford the high cost of the medication; another reason is that the drug was prescribed only to immunocompetent patients without comorbidities, a rare characteristic of those treated in a tertiary public hospital. This low IMQ prescription for MC is consistent with data published in the United States of America, where the IMQ prescription rate among 6.4 million consultations for MC was only 7.0%.^7^

However, it is important to remember that the removal of Molluscum contagiosum (MC) on the face or anogenital region, based on physical methods such as curettage, is also challenging. These procedures can cause physical and emotional impact, especially in patients younger than 18 years. Therefore, a topical home therapeutic option, such as the use of IMQ, becomes valuable for both patients and physicians.

The use of Imiquimod (IMQ) for the treatment of Molluscum contagiosum (MC) is widely discussed in the scientific literature, but there is still no consensus on its indication. Previous studies have shown divergences: while some authors report favorable results with IMQ use, demonstrating efficacy in selected cases,^2^, ^3^, ^7^ others question the actual drug effectiveness, stating that there are not enough randomized clinical trials to support it as an effective treatment for MC, especially when compared to conventional methods, such as curettage.^8^, ^9^, ^10^, ^11^ These divergent studies state that, in trials involving 470 children aged two to 12 years, the application of IMQ three times a week, for up to 16 weeks, did not result in a significant difference compared to control groups, with complete resolution rates of 24% (52/217) and 26% (28/106), respectively. Similar observations were made in another study, where the resolution rate was 24% (60/253) in the treated group, versus 28% (35/126) in the control group.^4^, ^8^, ^9^, ^10^, ^11^ However, it is worth noting that these studies did not evaluate IMQ efficacy in specific anatomical regions, such as the face and anogenital area, which may influence the overall conclusions. In the present study, when focusing on lesions located in areas with thin skin, a complete cure rate of 71.42% (15/21) of patients was observed, with 73.33% (11/15) of these cases achieving cure within 16 weeks.

However, the limited sample size should be taken into account, since the small number of treated patients influences the success rates. Furthermore, in four cases with a positive response after 16 weeks of treatment, it is important to consider the possibility of spontaneous involution of the lesions, a phenomenon observed in MC. Finally, one of the patients required 28 weeks (seven months) of treatment, reinforcing the importance of duration of use for therapeutic success.

The careful selection of patients and lesions to be treated with Imiquimod (IMQ) was essential for the positive results observed in the present study. Only lesions located in small areas of the face and anogenital region were treated, regions where the skin is thinner, which may have favored product absorption and local immune system activation, contributing to treatment effectiveness. In addition, the profile of the selected patients ‒ immunocompetent and without comorbidities ‒ also seems to have been decisive for therapeutic success, since these patients respond better to the immune stimulation provided by IMQ. These factors may explain the complete cure rate observed in 71.42% (15/21) of the cases, a more effective result than that reported in previous studies. Farhangian et al.^7^ also highlight that, although some studies show limited efficacy, the use of IMQ may be beneficial in localized diseases, especially in areas of thin skin, reinforcing the results observed in the present study. Therefore, the choice of the patient and the appropriate lesion, combined with the application in areas with characteristics that favor drug absorption, seem to be essential for the success of IMQ treatment in molluscum contagiosum.

Regarding imiquimod safety, five patients (23.8%) showed mild side effects, such as erythema (three cases) and impetigo (two cases). All adverse effects were quickly resolved with temporary drug discontinuation and, in cases of impetiginization, a brief course of topical antibiotic therapy was sufficient for control. These events are consistent with the literature findings, which generally indicate a favorable safety profile when used in limited areas and at adequate doses. Although rare, more severe adverse events, such as leukopenia or Stevens-Johnson syndrome, have been described in other studies but were not observed in this group of patients, reinforcing the relative safety of the treatment in selected and well-monitored populations.^3^, ^4^, ^12^

MC treatment is challenging; despite being a benign lesion, it is an infectious disease that is easily transmitted, which causes a psychological impact on patients and their families who seek a rapid, effective resolution with few side effects. Although IMQ is not the first-choice treatment in clinical practice, it is possible that its use in immunocompetent patients with MC located in regions where the epithelium is thinner may result in better cure rates. It is important to note that, as with all other treatments, the use of IMQ may lead to side effects, most of which are mild and reversible.

## Financial support

None declared.

## Authors’ contributions

Bruna Cavaleiro de Macedo Souza: Design and planning of the study; collection of data, or analysis and interpretation of data; statistical analysis; drafting and editing of the manuscript or critical review of important intellectual content; collection, analysis and interpretation of data; effective participation in research orientation; intellectual participation in the propaedeutic and/or therapeutic conduct of the studied cases; critical review of the literature; approval of the final version of the manuscript.

Rebeca Naomi Shida: Design and planning of the study; collection of data, or analysis and interpretation of data; statistical analysis; drafting and editing of the manuscript or critical review of important intellectual content; collection, analysis and interpretation of data; effective participation in research orientation; intellectual participation in the propaedeutic and/or therapeutic conduct of the studied cases; critical review of the literature; approval of the final version of the manuscript.

John Verrinder Veasey: Design and planning of the study; collection of data, or analysis and interpretation of data; statistical analysis; drafting and editing of the manuscript or critical review of important intellectual content; collection, analysis and interpretation of data; effective participation in research orientation; intellectual participation in the propaedeutic and/or therapeutic conduct of the studied cases; critical review of the literature; approval of the final version of the manuscript.

Guilherme Camargo Julio Valinoto: Design and planning of the study; collection of data, or analysis and interpretation of data; statistical analysis; drafting and editing of the manuscript or critical review of important intellectual content; collection, analysis and interpretation of data; effective participation in research orientation; intellectual participation in the propaedeutic and/or therapeutic conduct of the studied cases; critical review of the literature; approval of the final version of the manuscript.

## Conflicts of interest

None declared.
